# Domain-Adaptive Transfer Learning for HPV Lesion Classification in Whole Slide Images: A Patient-Level Pipeline Across the Cytology–Histology Continuum

**DOI:** 10.3390/bioengineering13060664

**Published:** 2026-06-05

**Authors:** Annabella Di Mauro, Pasquale De Luca, Maria Lina Tornesello, Emanuel Di Nardo, Luca D’Anna, Andrea Cerasuolo, Veronica Sanna, Saverio Simonelli, Vincenzo Gigantino, Antonella Gioioso, Margherita Cerrone, Rossella De Cecio, Gerardo Ferrara, Livia Marcellino, Angelo Ciaramella

**Affiliations:** 1 Pathology Unit, Istituto Nazionale Tumori IRCCS “Fondazione G. Pascale”, 80131 Naples, Italy; annabella.dimauro@istitutotumori.na.it (A.D.M.); v.sanna@istitutotumori.na.it (V.S.); saverio.simonelli@istitutotumori.na.it (S.S.); v.gigantino@istitutotumori.na.it (V.G.); a.gioioso@istitutotumori.na.it (A.G.); margherita.cerrone@istitutotumori.na.it (M.C.); r.dececio@istitutotumori.na.it (R.D.C.); gerardo.ferrara@istitutotumori.na.it (G.F.); 2 Department of Science and Technology, Università degli Studi di Napoli “Parthenope”, 80143 Naples, Italy; emanuel.dinardo@uniparthenope.it (E.D.N.); luca.danna001@studenti.uniparthenope.it (L.D.); livia.marcellino@uniparthenope.it (L.M.); angelo.ciaramella@uniparthenope.it (A.C.); 3 Molecular Biology and Viral Oncology Unit, Istituto Nazionale Tumori IRCCS “Fondazione G. Pascale”, 80131 Naples, Italy; m.tornesello@istitutotumori.na.it (M.L.T.); a.cerasuolo@istitutotumori.na.it (A.C.)

**Keywords:** deep learning, digital pathology, human papillomavirus, whole slide images, cytology–histology continuum, p16 immunohistochemistry, domain-adaptive transfer learning, SIPaKMeD, ResNet50, Macenko normalization, focal Loss, leave-one-patient-out cross-validation

## Abstract

The clinical translation of automated HPV detection in Whole Slide Images (WSIs) is challenged by staining variability, sparse viral effects, and the biological continuum between cytology and histology. This work presents a fully automated pipeline for binary patch-level classification of HPV-induced lesions on H&E-stained tissue. The core contribution is a domain-adaptive transfer learning strategy: a ResNet50 backbone is pretrained on the SIPaKMeD cervical cytology dataset rather than ImageNet, then fine-tuned on a target histological cohort. Preprocessing includes adaptive tissue segmentation, blur rejection, and Macenko stain normalization to ensure vendor-agnostic inputs. Evaluated using a strict Leave-One-Patient-Out cross-validation on 42 diagnostic specimens, the SIPaKMeD-based initialization significantly outperforms the ImageNet baseline. This approach achieves higher AUC-ROC scores and superior stability across folds, demonstrating that domain-specific pretraining effectively mitigates data scarcity and class imbalance in digital cervical cancer screening. Under a complementary 5-fold patient-level cross-validation covering all 19 patients of the cohort (133,704 patches, 7181 HPV-positive, prevalence 5.37%), the SIPaKMeD-pretrained model attains a mean test AUC-ROC of 0.694 with a 95% patient-aware bootstrap confidence interval of [0.681, 0.705], consistently above the ImageNet baseline mean of 0.656 obtained on the controlled three-fold ablation.

## 1. Introduction

Persistent infection by high-risk genotypes of Human Papillomavirus (HPV), most notably HPV-16 and HPV-18, is the established etiological agent of the vast majority of cervical carcinomas. The oncogenic mechanism is driven by the viral E6 and E7 proteins, which degrade the tumor-suppressor proteins p53 and pRb and disrupt cell-cycle control, leading to progressive epithelial dysplasia and, ultimately, to invasive disease. Although molecular HPV-DNA testing offers high analytical sensitivity for the detection of viral genomes, it does not discriminate between transient infections and clinically relevant precancerous lesions. Histopathological assessment of tissue biopsies therefore remains the diagnostic gold standard, based on the identification of specific morphological alterations such as koilocytosis and dyskeratosis. In this setting, immunohistochemical biomarkers such as p16 are frequently employed as surrogate indicators of HPV-driven oncogenic activity, particularly in diagnostically challenging or borderline lesions, further highlighting the multi-level nature of pathological assessment [1].

Importantly, these morphological alterations do not occur as isolated events but are distributed along a cytology–histology continuum, ranging from subtle cytological abnormalities to progressively disorganized epithelial architecture in high-grade lesions. This biological continuity represents a fundamental aspect of HPV-related disease and a critical challenge for both human interpretation and computational modeling [2]. The manual interpretation of histological slides is further limited by substantial inter-observer variability and by the inherently focal distribution of HPV cytopathic effects, which may be confined to small foci scattered within large regions of morphologically normal tissue. The digitization of glass slides into Whole Slide Images (WSI), multi-gigapixel, pyramidally structured files that reproduce the entire specimen at multiple magnifications, has opened the possibility of applying deep learning models, and in particular Convolutional Neural Networks (CNNs), to extract diagnostic features directly from pixel data [3,4].

Several recent studies have demonstrated the potential of deep learning for HPV-related histological analysis. Chakravarthy et al. [5] developed CNN-based models capable of predicting molecular subtypes of cervical cancer directly from histology, showing that WSIs carry morphological signatures correlated with viral infection. A systematic review and meta-analysis by Zhu et al. [6] reported area-under-the-curve (AUC) values above 0.85 for HPV-status prediction in oropharyngeal squamous cell carcinoma, identifying dataset heterogeneity as the principal obstacle to clinical translation. Schömig-Markiefka et al. [7] proposed vendor-agnostic pipelines for computational pathology and emphasized that automated tissue segmentation and quality control are prerequisites for scalable and reproducible models. Within this literature, three issues remain only partially addressed. First, extreme class imbalance, arising from the numerical prevalence of histologically healthy tissue over pathological foci, biases models toward the majority class when trained with standard cross-entropy. Second, chromatic variability induced by differences in staining protocols, reagent batches, fixation durations, and scanner calibrations can be exploited by the network as a spurious predictor if not explicitly normalized [8,9]. Third, the use of patch-level random splits in cross-validation allows patches from the same patient to appear both in training and test sets, producing inflated performance estimates that do not reflect generalization to unseen subjects. A further, often underexplored, limitation is that most current models do not explicitly account for the biological and diagnostic continuum underlying HPV-related lesions, thereby reducing their ability to capture transitional morphological patterns that are critical in routine pathology practice.

A less frequently discussed, yet increasingly important, aspect of model design in medical imaging is the choice of the source domain for transfer learning. The de facto standard is ImageNet initialization, but Raghu et al. [10] have shown that domain-specific pretraining on medical images can yield superior performance on medical targets, even when the intermediate dataset is much smaller than ImageNet. More recent work on self-supervised pretraining on medical corpora [11] further supports the intuition that reducing the domain gap between source and target tasks is a first-order design decision.

The present work builds on these observations and focuses on a specific research question: does domain-adaptive pretraining on the SIPaKMeD cervical cytology dataset [12] provide a measurable benefit for patch-level HPV classification in H&E-stained WSIs, under a strict patient-level evaluation protocol spanning the cytology–histology continuum? The contributions can be summarized as follows. A fully automated, vendor-agnostic preprocessing chain is implemented, combining Otsu thresholding on the HSV saturation channel, Laplacian-variance blur rejection, and Macenko stain normalization. A ResNet50 backbone [13] is then pretrained on SIPaKMeD, which shares morphological primitives with the target domain, and subsequently fine-tuned on the HPV cohort under Focal Loss [14] and weighted sampling to handle class imbalance. Evaluation is performed through a three-fold Leave-One-Patient-Out (LOPO) cross-validation that strictly forbids inter-patient information leakage. The SIPaKMeD-pretrained model is directly compared against an ImageNet-pretrained baseline on the same folds, providing a controlled quantification of the benefit of domain-adaptive initialization.

## 2. Materials and Methods

### 2.1. Dataset and Experimental Setting

**Figure 1:**
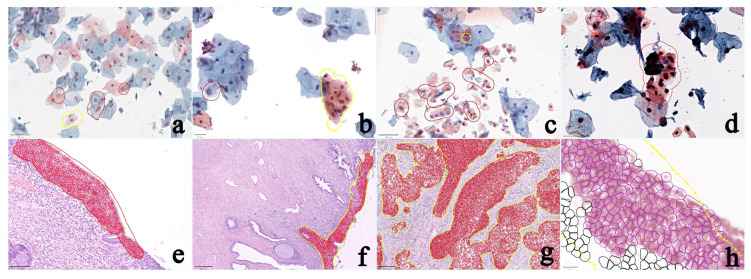
Cytology–histology continuum and multiscale annotation framework underlying the dataset. (**a**–**d**) Cytological spectrum from LSIL to HSIL, showing koilocytic changes, increasing nuclear atypia, and hyperchromasia; representative atypical cells are highlighted in red (diagnostically relevant) and yellow (ancillary). (**e**–**g**) Corresponding histological progression from CIN1 to CIN3 and invasive squamous cell carcinoma, with pathologist-annotated ROIs outlined. (**h**) Example of ROI annotation combined with automated cell segmentation, illustrating the multiscale analytical approach that underpins the derivation of patch-level labels in the present work.

The study cohort was assembled within a diagnostic workflow spanning from cytological screening to histological evaluation, and comprises a total of 42 diagnostic specimens derived from 19 unique anonymized patients. For each case, cytological assessment (Pap test) was available prior to biopsy, followed by histopathological examination of Hematoxylin and Eosin (H&E)-stained tissue sections, which were subsequently digitized into Whole Slide Images (WSIs) under the supervision of an expert pathologist at a cancer institute. Regions of interest (ROIs) were annotated by expert pathologists to capture HPV-related morphological alterations across the cytology–histology continuum, ensuring biologically meaningful and diagnostically relevant training labels, as illustrated in Figure 1. The 42-specimen total decomposes into 19 Pap cytology preparations (one per patient), 19 H&E-stained biopsy WSIs (one per patient), and 4 additional p16 immunohistochemistry (IHC) slides obtained for diagnostically borderline cases in which HPV-driven oncogenic activity required surrogate molecular confirmation. The complete mapping between specimens and patients is reported in Table 1. When grouped at the patient level, this many-to-one structure reflects the multi-level diagnostic framework in which a single subject may contribute multiple specimens at different diagnostic scales; the LOPO evaluation protocol described below operates on this patient-level grouping, so that no specimen-to-specimen correlation within a patient can leak across training and test partitions.

The H&E WSIs were tiled into 133,704 non-overlapping tissue patches of 256×256 pixels extracted at the highest magnification level (Level 0) of the WSI pyramid. Of these, 6181 (4.62%) are HPV-positive and 127,523 (95.38%) are HPV-negative, corresponding to a positive-to-negative ratio of approximately 1:20.6.

A subsequent comprehensive audit of the dataset metadata, performed at the patient level, yielded the verified class distribution adopted in all analyses reported in this work: 7181 HPV-positive patches (5.37%) and 126,523 HPV-negative patches, with a negative-to-positive ratio of 1:17.6. The complete per-patient breakdown is reported in Table 2, sorted by descending positive prevalence to make the heterogeneity of the cohort apparent. Six patients contain zero HPV-positive patches; these contribute to the training partitions of all folds in which they are not held out, but cannot serve as informative held-out test patients in isolation.

Because the biological variability associated with only 19 subjects poses a concrete risk of patient-specific overfitting, the cohort is organized into two disjoint groups. Three subjects (referred to as P1, P2, and P3) are designated as primary test patients and rotate through the three LOPO folds; the remaining sixteen subjects are used exclusively to augment the training partition across all folds. The composition of the primary patients is reported in Table 3. This design maximizes the utilization of the available patient-level variability during training while still enforcing patient-level separation at test time.

To complement the three-patient ablation rotation described above and provide an evaluation that covers every subject of the cohort, a second cross-validation scheme has been defined in which all 19 patients are partitioned into five disjoint folds. Each patient appears in exactly one test fold (four patients per fold, except fold 4 which contains three patients). The fold composition is reported in Table 4; the corresponding evaluation protocol is described in Section 2.4 and the results are reported in Section 3.7.

All experiments were performed on a workstation equipped with an NVIDIA RTX 4080 SUPER GPU (16 GB of dedicated memory), using Python 3.8+ with PyTorch, OpenSlide, OpenCV, NumPy, and the TIAToolbox library. No patient-identifying information was retained at any stage of the pipeline.

### 2.2. Preprocessing Pipeline

Given the multi-gigapixel nature of WSIs, a sequence of preprocessing operations is applied before training to isolate informative tissue regions, suppress low-quality content, and harmonize chromatic appearance across specimens. A synthetic comparison between the chosen techniques and standard alternatives in the literature is reported in Table 5.

**Figure 2:**
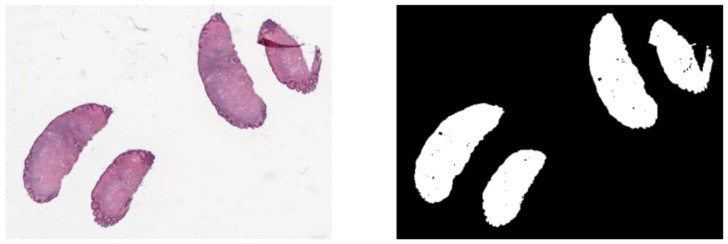
Automated tissue segmentation on a representative WSI. **Left**: RGB thumbnail. **Right**: binary tissue mask obtained by Otsu thresholding on the HSV saturation channel. White regions identify tissue foreground, black regions identify background.

Each WSI is first downsampled to a thumbnail representation and converted from RGB to the HSV color space. The saturation channel is used as the basis for tissue–background separation because stained tissue exhibits high saturation, whereas the glass-slide background is close to achromatic. The Otsu algorithm [15] is then applied to compute, in an unsupervised and scanner-agnostic way, the threshold $t^{*}$ that minimizes the intra-class variance

(1) $$\sigma_{w}^{2}\left(t\right)\;=\;\omega_{0}\left(t\right)\;\sigma_{0}^{2}\left(t\right)+\omega_{1}\left(t\right)\;\sigma_{1}^{2}\left(t\right),$$

where $\omega_{0}\left(t\right)$ and $\omega_{1}\left(t\right)$ denote the probabilities of the two classes (background and tissue) separated by threshold *t*, and $\sigma_{0}^{2}\left(t\right)$, $\sigma_{1}^{2}\left(t\right)$ their within-class variances. The resulting binary mask is used to guide high-resolution patch extraction: for each candidate 256×256 tile, coordinates are mapped back onto the mask, and the tile is retained only if at least 75% of its area corresponds to foreground tissue. A qualitative example of tissue segmentation is shown in Figure 2.

**Figure 3:**
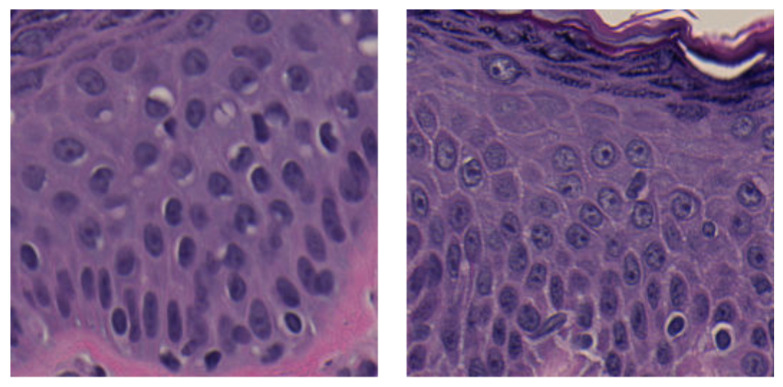
Laplacian-variance quality control. A blurred patch (**left**, variance below threshold) is rejected, while a sharp patch (**right**, variance above threshold) is retained.

Each retained patch is subsequently subjected to blur quality control. Image sharpness is quantified as the variance of the Laplacian response $\Delta I=\partial^{2}I/\partial x^{2}+\partial^{2}I/\partial y^{2}$ computed on the grayscale version of the patch: this quantity is large when the image contains well-defined edges (nuclear membranes, cellular borders) and small when the patch is out of focus. Patches with a Laplacian variance below a fixed threshold T=100 are discarded. On the reference CMU-1 slide adopted as a methodological sanity check, this filter rejected 2.45% of the extracted tiles (5 out of 204) while retaining 97.55% as sharp. Representative examples of accepted versus rejected patches are reported in Figure 3.

**Figure 4:**
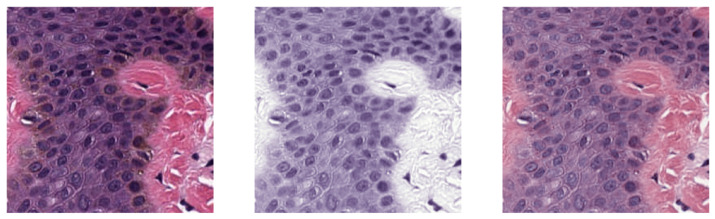
Macenko stain normalization. **Left**: original patch. **Center**: extracted Hematoxylin channel. **Right**: Macenko-normalized patch mapped onto the shared reference appearance.

The final preprocessing step is chromatic standardization via the Macenko stain-normalization algorithm [8]. Unlike purely statistical approaches such as Reinhard normalization, which operates on generic channel statistics, Macenko’s method is physically motivated: pixel intensities are first mapped to the optical density (OD) domain according to the Beer–Lambert law

(2) $$\mathrm{OD}\;=\;-\log_{10}\;\left(\frac{I}{I_{0}}\right),$$

where *I* is the pixel intensity and $I_{0}$ the background illumination. Singular Value Decomposition (SVD) is then applied to identify the principal stain-vector directions corresponding to Hematoxylin and Eosin, and each patch is reprojected onto a shared reference appearance. The effect of this transformation is illustrated in Figure 4. This stage suppresses chromatic variations arising from reagent batches, fixation conditions, and scanner calibrations [9].

### 2.3. Domain-Adaptive Transfer Learning and Training Protocol

The classification model is based on a ResNet50 backbone [13], whose default classification head is replaced by a task-specific module composed of a dropout layer (p=0.5), a fully connected layer projecting the 2048-dimensional feature vector to 512 units with ReLU activation, a second dropout layer (p=0.3), and a final linear layer producing a single sigmoid-activated logit.

Rather than initializing the backbone with generic ImageNet weights, a domain-adaptive transfer-learning strategy is adopted. The ResNet50 backbone is first pretrained in a binary classification task on the SIPaKMeD dataset [12], a publicly available corpus of 4049 Pap-smear cytological images organized into five morphological classes. The task is cast as koilocytotic vs. non-koilocytotic binary classification: the single class koilocytotic cells (825 images) is treated as positive, and the remaining four classes (superficial–intermediate, parabasal, metaplastic, dyskeratotic; 3224 images in total) are grouped as negative. The rationale is that koilocytes are the cytopathic hallmark of HPV infection, and a backbone that has already learned to localize koilocytotic features in a cytological context is expected to provide a more informative initialization for the target histological task than a backbone trained on natural images, consistently with the cytology–histology continuum underlying HPV-related disease. Pretraining is performed for 50 epochs with the AdamW optimizer [16] and Focal Loss, and the best-validation checkpoint is retained for fine-tuning.

Fine-tuning on the target HPV cohort is then performed as an end-to-end update of the full ResNet50 network, starting from the SIPaKMeD-pretrained weights. The learning rate is set to $1\times 10^{-4}$, an order of magnitude lower than the one used for SIPaKMeD pretraining, so as to refine the inherited representations without erasing them. The model is trained for 50 epochs with a cosine-annealing schedule using the AdamW optimizer (weight decay 0.01). The main hyperparameters are summarized in Table 6.

Class imbalance is addressed by two complementary mechanisms. First, the training loss is the Focal Loss [14],

(3) $$\mathrm{FL}\left(p_{t}\right)\;=\;-\alpha_{t}\;{\left(1-p_{t}\right)}^{\gamma }\;\log\left(p_{t}\right),$$

where $p_{t}$ denotes the predicted probability of the ground-truth class, $\alpha_{t}$ is a class-balancing weight (set to α=0.75 for the positive class), and γ=2.0 is the focusing parameter. The modulating factor ${\left(1-p_{t}\right)}^{\gamma }$ down-weights the contribution of easily classified examples and drives the optimization toward the morphologically ambiguous positive patches. Second, a WeightedRandomSampler with inverse-frequency weights is used during mini-batch construction, so that each batch contains a meaningful number of minority-class examples.

Online data augmentation during training comprises random resized crops (scale 0.8–1.0 to 224×224), random horizontal and vertical flips, random rotations in $\left[-15^{\circ },+15^{\circ }\right]$, and mild color jitter (brightness ±0.2, contrast ±0.2, saturation ±0.1, hue ±0.05). Validation and test patches are processed with a deterministic resize-and-center-crop pipeline to guarantee reproducible evaluation, and all images are normalized with ImageNet channel statistics (μ=[0.485, 0.456, 0.406], σ=[0.229, 0.224, 0.225]) to retain compatibility with the pretrained backbone.

### 2.4. Evaluation Protocol

Model evaluation is performed under a patient-level Leave-One-Patient-Out (LOPO) cross-validation scheme with three folds, one for each primary test patient (P1, P2, P3). In each fold, all patches belonging to the held-out primary patient constitute the test set, while patches from the two remaining primary patients and from all sixteen training augmentation patients are used for training and validation through a further patient-level split, so that no patient, and, by construction, no specimen originating from a given patient within the 42-case collection, contributes patches to more than one partition. This design eliminates inter-patient information leakage by construction and provides an estimate of generalization to subjects genuinely unseen during training.

For each fold, the following metrics are computed on the test set: the Area Under the Receiver Operating Characteristic Curve (AUC-ROC), the Average Precision (AP), the best F1-score over decision thresholds in [0.05, 0.95] with a step of 0.05, and the corresponding sensitivity and specificity. Fold-level results are aggregated into mean and standard deviation (with n−1 degrees of freedom). To isolate the effect of domain-adaptive pretraining, the entire LOPO protocol is repeated twice under identical conditions: once with the backbone initialized from standard ImageNet weights, and once with the backbone initialized from the SIPaKMeD-pretrained checkpoint.

To provide an evaluation that covers every subject of the cohort and to avoid any selection bias in the choice of test patients, the SIPaKMeD-pretrained model is additionally evaluated under a 5-fold patient-level cross-validation that partitions the 19 patients into five disjoint folds (Table 4). In each fold, all patches belonging to the held-out patients constitute the test set, while patches from the remaining patients are used for training and validation through a further patient-level split. By construction, no patch and no specimen originating from a given patient appears in more than one partition; the leakage-free property of the Leave-One-Patient-Out (LOPO) principle is therefore preserved. The acronym LOPO is hereafter used in its general leakage-free sense; the specific protocols of this work are referred to as three-fold ablation (Section 3.3 and Section 3.4, Table 7 and Table 8) and 5-fold patient-level CV (Section 3.7 and following), respectively.

**Figure 5:**
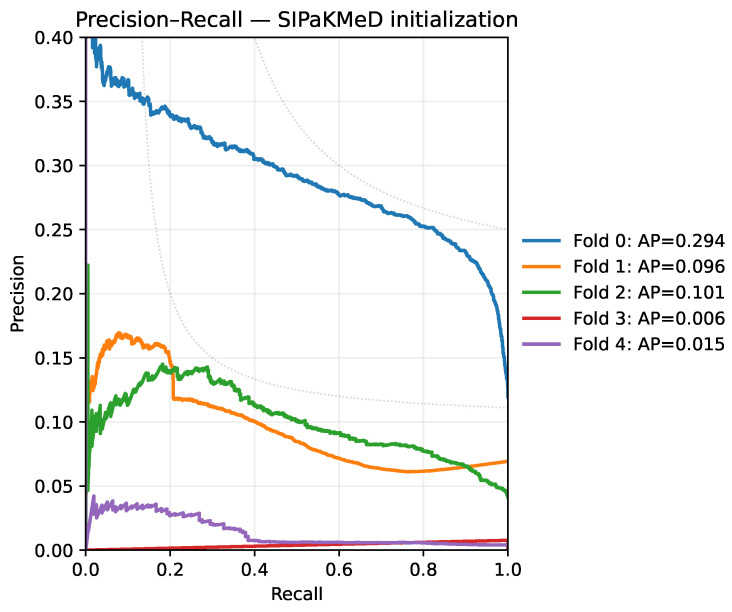
Precision–recall curves of the SIPaKMeD-pretrained model on each of the five test folds of the patient-level cross-validation. Dotted contours correspond to iso-$F_{1}$ levels. The Average Precision varies substantially across folds, from 0.294 on fold 0 (the fold with the highest positive support) down to 0.006 on fold 3, whose test set contains only two HPV-positive patches out of 947. This dispersion is intrinsic to Average Precision under extreme positive-class scarcity, and the AUC-ROC remains the most stable aggregate metric for cross-fold comparison.

In addition to the metrics enumerated above, the 5-fold analysis reports the Matthews Correlation Coefficient (MCC) at threshold 0.5, the balanced accuracy at threshold 0.5, the Brier score for probability calibration, the sensitivity at fixed specificity (≥90% and ≥95%), and the precision–recall curves of the SIPaKMeD-pretrained model on each test fold (Figure 5). The aggregate mean test AUC is further accompanied by a patient-aware non-parametric bootstrap 95% confidence interval with B=1000 resamples, which captures within-fold sampling variability while respecting the patient-level design. Checkpoint selection follows the standard best-validation-AUC rule throughout; the noise of this criterion under low positive support, already noted in Section 3.5, motivates more robust strategies (e.g., exponential moving average of late parameters or logit ensembling of late checkpoints), which we leave as future work.

## 3. Results

### 3.1. Preprocessing Outcomes

The preprocessing pipeline was preliminarily validated on the publicly available reference slide CMU-1 (46,000×32,914 pixels, nine pyramidal levels), used as a technical sanity check before being applied to the full cohort. Otsu thresholding on the HSV saturation channel produced a clean tissue mask without manual intervention, as shown in Figure 2. From this reference WSI, 204 candidate patches were extracted at Level 0, of which 199 (97.55%) passed the Laplacian-variance quality filter at T=100, and 5 (2.45%) were rejected as blurred. The same chain was applied to the 19 H&E WSIs of the study cohort (one per patient, obtained from the 42-specimen collection through patient-level grouping), yielding the final dataset of 133,704 tissue patches used for training and evaluation.

### 3.2. SIPaKMeD Pretraining

The intermediate SIPaKMeD pretraining task converged with high validation performance, reaching an AUC of 0.996 on the held-out 20% split of the cytology dataset. This confirms that the backbone had successfully learned to discriminate koilocytotic from non-koilocytotic cells before being transferred to the histological target, and produced the initialization checkpoint used in all subsequent HPV experiments.

### 3.3. Cross-Validation Performance

**Figure 6:**
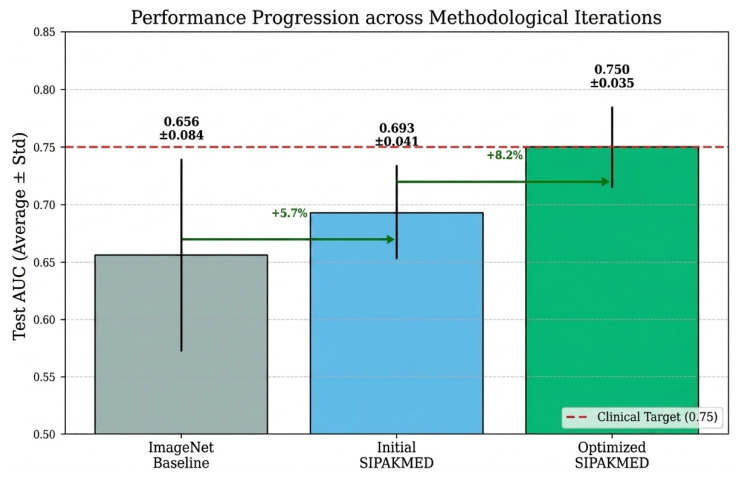
Training dynamics on a representative LOPO fold. Training loss, training AUC, and validation AUC are plotted as a function of the epoch.

**Figure 7:**
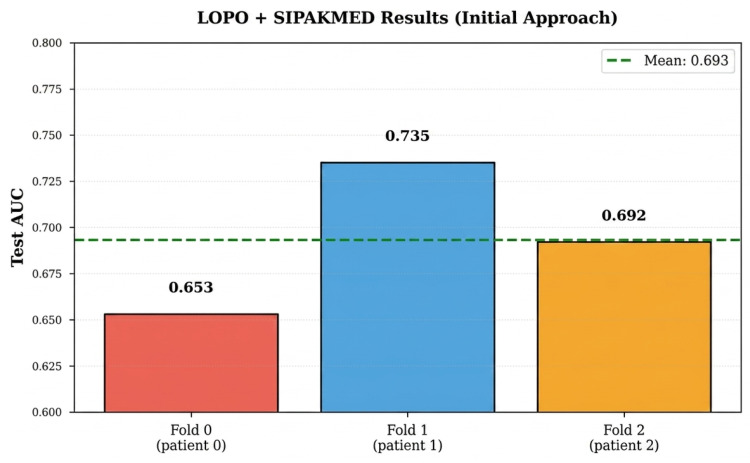
Per-fold test AUC of the SIPaKMeD-pretrained model across the three LOPO folds. The dashed line marks the aggregate mean.

The aggregate performance of the pipeline across the three LOPO folds is summarized in Table 7 and visualized in Figure 6 and Figure 7. With SIPaKMeD-pretrained initialization, the model achieves a mean test AUC-ROC of 0.693±0.041, with per-fold values ranging from 0.653 (P1) to 0.735 (P2). The mean Average Precision is 0.285±0.239 and the mean best F1-score is 0.275±0.161. The mean best validation AUC is 0.652±0.136; as discussed below, the fact that validation AUC is lower and more variable than test AUC is a direct consequence of the extremely small number of positive samples in the per-fold validation sets (between 22 and 32 positive patches), which makes the validation metric statistically noisy.

### 3.4. Impact of Domain-Adaptive Pretraining

To quantify the specific benefit of SIPaKMeD pretraining, the LOPO protocol was repeated with an ImageNet-initialized backbone while keeping every other component (preprocessing, head architecture, optimizer, loss, sampling, augmentation, evaluation) identical. The comparison is reported in Table 8. SIPaKMeD pretraining improves the mean test AUC from 0.656 to 0.693 (+3.7 absolute points, +5.7% relative) and, notably, more than halves the per-fold standard deviation (from 0.084 to 0.041). This reduction of variance indicates that domain-adaptive pretraining produces a more stable predictor across patients with highly different positive-class distributions, and is consistent with the observations of Raghu et al. [10] and Azizi et al. [11] on the benefits of domain-specific initialization for medical imaging tasks.

### 3.5. Training Dynamics and Validation Behavior

The training dynamics shown in Figure 6 exhibit a consistent downward trajectory of the training loss, with the training AUC climbing monotonically to values close to 0.95. In contrast, the validation AUC exhibits pronounced epoch-by-epoch oscillations. The cause of this behavior can be quantitatively attributed to the composition of the validation partitions: the number of positive examples per validation fold is 22 (Fold 0), 32 (Fold 1), and 32 (Fold 2), respectively, against thousands to tens of thousands of negative examples. Under such extreme imbalance, the validation AUC is dominated by statistical noise rather than by meaningful generalization trends, which explains why, at the aggregate level, the mean validation AUC (0.652) is lower and more variable than the mean test AUC (0.693). This observation also motivates caution in interpreting the validation metric as a reliable criterion for early stopping or checkpoint selection in this setting.

### 3.6. Interpretation of Imbalance-Sensitive Metrics

The Average Precision and F1-score display a large fold-to-fold variability (±0.239 and ±0.161 respectively), driven almost entirely by Fold 2 (patient P3), whose test set contains only 1.0% of positive patches. Under such extreme imbalance, even a small absolute number of false positives collapses precision, which in turn depresses both AP and F1 through their harmonic or integral dependence on precision. The AUC-ROC, which evaluates ranking ability across all decision thresholds, is less sensitive to this regime and is the most appropriate summary metric for the problem at hand.

### 3.7. Evaluation Across the Full Cohort: 5-Fold Patient-Level Cross-Validation

The SIPaKMeD-pretrained model has been re-evaluated under the 5-fold patient-level cross-validation protocol described in Section 2.4, which covers all 19 patients of the cohort in five disjoint test folds (Table 4). The per-fold and aggregate metrics are reported in Table 9; the precision–recall curves on each fold are shown in Figure 5.

### 3.8. Robustness: Bootstrap Confidence Interval

To quantify the statistical uncertainty associated with the 5-fold mean test AUC reported in Table 9, a non-parametric patient-aware bootstrap with B=1000 resamples has been computed. In each bootstrap iteration, patches within each test fold are resampled with replacement (a cluster bootstrap, since each fold owns a distinct subset of patients), and the per-fold AUC is recomputed and averaged. The procedure captures within-fold sampling variability conditional on the observed patient-level fold composition while respecting the leakage-free patient-level design.

The resulting 95% bootstrap confidence interval on the mean test AUC-ROC of the SIPaKMeD-pretrained model is [0.681, 0.705], centered on the point estimate 0.694 (Table 10). This interval lies entirely above the ImageNet baseline mean of 0.656 reported in Table 8, supporting the consistency of the domain-adaptive pretraining benefit. We report this interval as descriptive evidence of variability and reproducibility, without invoking a hypothesis test on the difference, since with n=5 folds any aggregate paired test would have very limited statistical power.

### 3.9. Comparison with Classical Machine-Learning Baselines

To place the deep learning result in the context of non-CNN approaches, two classical baselines have been trained under the same 5-fold patient-level cross-validation protocol: an $\ell_{2}$-regularized Logistic Regression (LR) and an XGBoost classifier. Both operate on a 70-dimensional handcrafted descriptor of each patch, which combines per-channel statistical moments on RGB and HSV, saturation/value/hue histograms, gradient-magnitude statistics, and a downsampled block-variance texture feature. The descriptor is designed to be lightweight and reproducible and is deliberately kept free of dataset-specific tuning. Fold 3 is excluded from the aggregate statistics because its test set contains only two HPV-positive patches, making the per-fold AUC and AP estimates unstable; the aggregate is therefore computed on the remaining four folds.

The results are reported in Table 11. The $\ell_{2}$-regularized Logistic Regression on handcrafted features attains a mean AUC of 0.736, and XGBoost a mean AUC of 0.713; both are competitive with the SIPaKMeD-pretrained CNN (0.686 on the same four folds). A closer inspection reveals that the classical models obtain markedly higher AUC on fold 1 (LR: 0.893; XGBoost: 0.897; CNN: 0.530), which contains the patient with the highest HPV-positive prevalence (40.16%), while the CNN dominates on the other folds. This pattern is consistent with the interpretation that a high-capacity deep model can be more susceptible to overfitting on patient-specific tissue cues when a single outlier patient strongly drives the training–test relationship, whereas a low-capacity classifier on engineered features is more conservative under such a regime. The deep representation nonetheless retains downstream advantages that classical baselines cannot easily offer, including direct compatibility with attention-based Multiple Instance Learning aggregation, end-to-end fine-tuning without manual feature design, and visual interpretability via Grad-CAM; these advantages motivate its continued use as the primary modelling approach of this work.

### 3.10. Patient-Level Aggregation: Case Study

The metrics reported in the previous subsections are computed at the patch level, which is the granularity at which the model operates. A clinical decision support workflow, by contrast, requires patient-level or slide-level outputs. To explore the translation of patch-level probabilities into patient-level scores, a preliminary case study has been conducted on the five held-out test folds of the patient-level cross-validation. For each fold, the patch-level posterior probabilities of the SIPaKMeD-pretrained model are aggregated into a single per-fold risk score using three rules: (i) mean probability across all patches in the fold; (ii) mean of the top-10 patches by predicted probability; (iii) the 95th percentile of the per-fold probability distribution.

The aggregated scores are reported in Table 12 together with the ground-truth fraction of HPV-positive patches in each fold. Because each test fold contains only 3 or 4 patients, the analysis is presented as a case study rather than as a statistically robust patient-level benchmark: a formal patient-level AUC would require substantially more test patients per fold. The reported scores illustrate that simple aggregation rules already generate clinically interpretable per-fold signals from the patch-level model output, providing a concrete starting point for downstream prioritization or triage workflows. A formal patient-level evaluation via attention-based Multiple Instance Learning on a larger multi-center cohort is the natural next step and is identified as the primary clinically oriented future direction.

## 4. Discussion

The main empirical finding of this study is that replacing ImageNet pretraining with SIPaKMeD pretraining produces a measurable and reproducible improvement on a small-cohort HPV histopathology task. The improvement in mean test AUC from 0.656 to 0.693 is moderate in absolute terms, but is obtained under a strict LOPO evaluation and is accompanied by a halving of the per-fold variance, indicating that the SIPaKMeD-initialized model generalizes more uniformly across patients with very different disease prevalence and morphological characteristics. Three aspects of this result deserve explicit discussion.

The first aspect concerns the motivation for domain-adaptive pretraining. ImageNet and histopathology differ substantially in low- and mid-level image statistics, and several recent works have pointed out that the benefit of ImageNet initialization in medical imaging, although non-zero, is smaller than commonly assumed [10,11]. SIPaKMeD, although a cytological rather than histological dataset, shares with the target domain the morphological primitives that are most discriminative for HPV-associated alterations, namely nuclear shape, chromatin texture, and perinuclear halos. The high validation AUC (0.996) achieved on the binary koilocytotic versus non-koilocytotic subtask of SIPaKMeD confirms that the backbone has acquired an explicit representation of the cytopathic hallmark of HPV before being exposed to the histological target.

The second aspect is the strict enforcement of patient-level partitioning. A relevant fraction of the existing literature on patch-based classification in digital pathology reports performance estimates obtained through random, patch-level splits, which allow patches from the same patient to appear in both training and test sets. The LOPO scheme adopted here guarantees that each test fold is evaluated on tissue from a patient never seen during training, at the cost of increased per-fold variance. In this context, the reduction of variance from 0.084 to 0.041 observed when moving from ImageNet to SIPaKMeD pretraining is particularly meaningful: it suggests that domain-adaptive initialization mitigates the sensitivity of the model to inter-patient variability, which is one of the main obstacles to clinical translation identified by Zhu et al. [6].

Beyond its technical implications, the observed improvement in generalization stability has direct translational relevance. In routine cervical pathology, diagnostic variability, particularly in borderline categories such as LSIL versus HSIL, remains a well-recognized challenge, with implications for patient management and follow-up strategies. A model that is less sensitive to inter-patient variability, as suggested by the reduced variance observed with SIPaKMeD pretraining, may contribute to more consistent decision support across heterogeneous clinical scenarios. From a biological perspective, the ability of the model to capture cytopathic features associated with HPV infection, such as koilocytic changes and nuclear atypia, supports its potential role in bridging cytology and histology within a unified analytical framework. This is particularly relevant in screening settings, where cytological and histological assessments are inherently complementary but often disconnected in digital workflows. In this context, domain-adaptive pretraining may represent a step toward more clinically aligned artificial intelligence systems, capable not only of improving classification performance but also of reflecting the underlying disease biology. Future integration with slide-level aggregation and explainability tools could further enhance clinical interpretability and facilitate adoption in diagnostic practice, in line with recent efforts to translate AI models into real-world pathology workflows [17,18].

The third aspect is the role of preprocessing. Macenko normalization, Otsu segmentation, and Laplacian-variance quality control are often treated as implementation details, yet they directly affect what the model is allowed to learn. The proposed chain is fully automatic, free from manually selected thresholds, and consistent with the vendor-agnostic design advocated by Schömig-Markiefka et al. [7] and by Tellez et al. [9]. It therefore provides a reproducible entry point for multi-center extensions of this work.

Several limitations must be acknowledged. The cohort of 19 patients (assembled from 42 diagnostic specimens spanning Pap cytology, H&E biopsy, and p16 IHC) is small by deep learning standards, and the three-fold LOPO design, while rigorously leakage-free, produces wide confidence intervals simply because n=3. The single-center nature of the data prevents a direct quantification of the vendor-agnostic claims associated with Macenko normalization. The validation sets are severely imbalanced and contain only a few tens of positive samples, which renders the validation AUC a noisy model selection criterion and motivates more robust checkpoint selection strategies as a direction for future work. The model operates at the patch level and does not yet aggregate predictions into patient- or slide-level diagnoses, which would be the natural next step through Multiple Instance Learning (MIL) formulations [19]. Finally, the absence of explainability mechanisms such as Grad-CAM [20] limits the interpretability of predictions for clinical end-users.

A direct numerical comparison with recent HPV-prediction studies is not straightforward, since the reported AUC values above 0.85 are typically obtained on oropharyngeal rather than cervical tissue, on slide-level rather than patch-level labels, and on larger, multi-institutional cohorts with MIL aggregation [6]. The patch-level AUC of 0.693 reported here, obtained under strict LOPO on a single-center H&E dataset, targets a more demanding evaluation scenario and is expected to constitute a lower bound with respect to patient-level or slide-level formulations.

Future work will focus on four main directions. First, the cohort will be expanded through multi-center collaboration, which is the primary lever to reduce the per-fold variance that currently dominates the uncertainty of the estimate. Second, the training protocol will be extended along two axes: a two-phase fine-tuning schedule, in which the backbone is initially frozen so that the classification head can adapt without perturbing the SIPaKMeD-derived representations, and a dual-checkpoint testing procedure designed to mitigate the unreliability of best-validation model selection under extreme imbalance. Third, Test-Time Augmentation over geometric transforms will be investigated as an inexpensive ensemble mechanism exploiting the rotational symmetry of histological tissue. Fourth, attention-based MIL will be integrated to aggregate patch-level scores into patient- or slide-level decisions, and explainability via Grad-CAM will be added to make the predictions visually inspectable by pathologists.

A complementary set of observations follows from the analyses reported in Section 3.7, Section 3.8, Section 3.9 and Section 3.10. The mean test AUC obtained under the 5-fold patient-level cross-validation (0.694, 95% bootstrap CI [0.681, 0.705]) is essentially identical to the value obtained on the controlled three-fold ablation rotation (0.693), providing direct empirical evidence that the SIPaKMeD pretraining benefit is robust to the choice of cross-validation design. The larger per-fold standard deviation under the 5-fold protocol (0.105 versus 0.041) is a faithful reflection of inter-patient heterogeneity in the full cohort, not a degradation of the model: when every patient is allowed to enter the test set, including patients whose tissue morphology departs substantially from the rest of the cohort, the fold-level performance unavoidably spans a wider range. This is, in our view, the most honest reading of generalization variability that a cohort of 19 subjects can produce under leakage-free evaluation.

The competitive performance of the classical XGBoost baseline on fold 1, which contains the patient with the highest HPV-positive prevalence (40.16%), deserves explicit comment. The CNN attains an AUC of 0.530 on this fold while XGBoost reaches 0.897. We interpret this gap as a consequence of the fact that a single outlier patient with an unusually high positive support can dominate the loss landscape of a high-capacity model and produce overfitting on patient-specific tissue cues, while a low-capacity classifier on engineered features is intrinsically more conservative under the same regime. Two practical mitigations suggest themselves: an ensemble that combines the deep representation with the handcrafted descriptor, and patient-stratified mini-batch sampling at training time. Both are identified as future directions.

Regarding the domain relationship between Pap-smear cytology and H&E histology, we note that, although the two modalities differ substantially in data appearance and structure (isolated cells on sparse background versus densely packed tissue architecture), the cytopathic hallmark of HPV infection (koilocytosis: perinuclear halos, irregular nuclear membranes, hyperchromasia) is morphologically conserved across the two modalities at the sub-cellular scale. The high validation AUC (0.996) achieved by the SIPaKMeD pretraining on its binary koilocytotic-versus-non-koilocytotic task indicates that this shared substrate is effectively acquired by the backbone, and the consistency of the downstream fine-tuned model under both the three-fold and the 5-fold patient-level protocols indicates that the substrate transfers effectively to the histological target. A quantitative feature-space alignment study (e.g., Centered Kernel Alignment between SIPaKMeD-domain and biopsy-domain activations) would provide additional insight into the structure of this transfer and is identified as a future direction.

On the methodological choice between supervised domain-adaptive transfer learning and self-supervised pretraining directly on the biopsy data, three considerations motivated the present approach. First, contemporary contrastive self-supervised frameworks display diminishing returns below approximately $10^{5}$ in-domain images per ResNet-class backbone [11], while our cohort yields 133,704 patches but only 19 unique patients, so the effective diversity is markedly lower than the patch count suggests. Second, contrastive objectives treat random pairs of crops as negatives; with a global positive prevalence of 5.37%, the negative pool is overwhelmingly dominated by visually similar healthy-tissue patches, a regime in which the embedding space can collapse around features unrelated to HPV cytopathic effects. Third, the SIPaKMeD source provides supervised labels for the canonical cytopathic hallmark of HPV (koilocytes), so the supervised signal is directly aligned with the downstream task in a way that self-supervised pretraining cannot, by construction, replicate. Self-supervised pretraining on a substantially larger multi-center biopsy corpus remains an attractive complementary direction for the future.

Finally, the patient-level aggregation case study (Section 3.10) confirms that simple rules can convert patch-level probabilities into clinically interpretable patient-level scores, but the absence of statistical power at the patient level (3–4 test patients per fold) reinforces the need for a formal patient-level evaluation through attention-based Multiple Instance Learning on a larger multi-center cohort, which we have already identified as the primary clinically oriented future direction.

## 5. Conclusions

This work has presented a fully automated, end-to-end deep learning pipeline for patch-level classification of HPV-associated lesions in H&E-stained Whole Slide Images, operating across the cytology–histology continuum of HPV-related disease. The pipeline integrates a vendor-agnostic preprocessing chain, Otsu segmentation on the HSV saturation channel, Laplacian-variance quality control, and Macenko stain normalization, with a ResNet50 classifier subjected to domain-adaptive transfer learning from the SIPaKMeD cytology dataset. Class imbalance is addressed through Focal Loss and a weighted random sampler, and the model is evaluated under a strict three-fold Leave-One-Patient-Out protocol over 42 diagnostic specimens grouped into 19 unique anonymized patients and 133,704 patches. The SIPaKMeD-pretrained model attains a mean test AUC-ROC of 0.693±0.041, improving over an otherwise-identical ImageNet-initialized baseline (0.656±0.084) by +5.7% in mean AUC and, crucially, halving the per-fold variance. These results are obtained under no-leakage conditions and constitute a reproducible, honest baseline for HPV histopathology in small-cohort settings. The modular design of the pipeline naturally accommodates future extensions, including multi-center validation, refined fine-tuning schedules, Test-Time Augmentation, Multiple Instance Learning for patient-level aggregation, and explainability integration. These findings align with broader efforts to integrate artificial intelligence within multi-layered oncological frameworks, where computational models are expected to capture both morphological and molecular complexity [21].

Under the complementary 5-fold patient-level cross-validation that covers every subject of the cohort (Section 2.4 and Section 3.7), the SIPaKMeD-pretrained model attains a mean test AUC-ROC of 0.694 with a 95% patient-aware bootstrap confidence interval of [0.681, 0.705], in close agreement with the controlled three-fold ablation result and consistently above the ImageNet baseline mean of 0.656. The classical machine-learning baselines evaluated under the same protocol (Section 3.9) confirm that handcrafted-feature classifiers remain competitive in this small-cohort regime, while the deep representation retains the structural advantages that motivate its use as the primary modelling approach. The verified class distribution of the cohort (7181 HPV-positive patches out of 133,704, prevalence 5.37%, ratio 1:17.6) is the reference for all numerical claims of this work.

## Figures and Tables

**Table 1:** Decomposition of the 42 diagnostic specimens collected for the study into the three specimen types, and their grouping into 19 unique patients after patient-level aggregation. The downstream deep learning pipeline is trained and evaluated exclusively on patches extracted from the 19 H&E WSIs; the cytology and p16 IHC specimens provide diagnostic context and label verification at the case level.

Specimen Type	Specimens	Unique Patients	Role in Pipeline
Pap cytology preparation	19	19	Diagnostic context (label derivation)
H&E histology WSI	19	19	Image data for CNN training/testing
p16 IHC (borderline cases)	4	4	Surrogate oncogenic marker (label verification)
Total	42	19	—

**Table 2:** Per-patient breakdown of the 19-patient HPV cohort derived from the dataset audit. Patient identifiers are anonymized as $P_{01},\ldots ,P_{19}$, sorted by descending HPV-positive prevalence.

Patient	Patches	HPV+	HPV−	Prevalence
$P_{01}$	10,532	4230	6302	40.16%
$P_{02}$	11,534	1788	9746	15.50%
$P_{03}$	6984	372	6612	5.33%
$P_{04}$	619	22	597	3.55%
$P_{05}$	4776	98	4678	2.05%
$P_{06}$	1560	32	1528	2.05%
$P_{07}$	129	2	127	1.55%
$P_{08}$	56,636	568	56,068	1.00%
$P_{09}$	1608	4	1604	0.25%
$P_{10}$	2372	5	2367	0.21%
$P_{11}$	19,610	38	19,572	0.19%
$P_{12}$	1150	2	1148	0.17%
$P_{13}$	14,016	20	13,996	0.14%
$P_{14}$–$P_{19}$	2178	0	2178	0.00%
Total	133,704	7181	126,523	5.37%

**Table 3:** Composition of the three primary test patients used in the LOPO rotation. The remaining 16 patients contribute 55,002 patches used exclusively for training augmentation across all folds.

Patient	Patches	HPV-Positive	Positive Rate
P1	10,532	5788	55.0%
P2	11,534	1788	15.5%
P3	56,636	568	1.0%
Primary subtotal	78,702	8144	10.3%

**Table 4:** Composition of the five patient-level cross-validation folds covering the entire 19-patient cohort. Every patient appears in exactly one test fold; no patch is shared between training and test partitions.

Fold	Patients	Patches	HPV+	Prevalence	Range
0	4	15,183	1815	11.95%	0.00–15.50%
1	4	69,370	4802	6.92%	0.00–40.16%
2	4	9802	406	4.14%	0.00–5.33%
3	4	947	2	0.21%	0.00–1.55%
4	3	38,402	156	0.41%	0.14–2.05%
Total	19	133,704	7181	5.37%	0.00–40.16%

**Table 5:** Comparison between standard preprocessing strategies in computational pathology and the choices adopted in this work.

Challenge	Standard Methods	Adopted Approach
Chromatic variability	Reinhard/Macenko normalization	Macenko (Beer–Lambert + SVD)
Background/noise	Fixed-threshold segmentation	Adaptive Otsu on HSV saturation
Image quality	Manual patch curation	Laplacian variance (T=100)
Class imbalance	Class weighting/oversampling	Focal Loss + weighted sampler
Evaluation integrity	Random patch-level splits	Strict LOPO patient-level CV

**Table 6:** Summary of training hyperparameters for the fine-tuning stage on the HPV cohort.

Parameter	Value
Backbone	ResNet50
Pretraining source	SIPaKMeD cervical cytology dataset
Input size	224×224 (cropped from 256×256 patches)
Batch size	32
Epochs	50
Learning rate	$1\times 10^{-4}$
Optimizer/scheduler	AdamW (λ=0.01)/cosine annealing
Loss function	Focal Loss (α=0.75, γ=2.0)
Class imbalance sampler	WeightedRandomSampler (inverse-frequency)
Checkpoint selection	Best validation AUC

**Table 7:** Per-fold and aggregate LOPO cross-validation performance of the SIPaKMeD-pretrained model (n=3 folds). Aggregate statistics are reported as mean ± sample standard deviation.

Fold (Held-Out Patient)	Test AUC-ROC	Test AP	Test F1	Best Val AUC
Fold 0 (P1)	0.653	0.525	0.427	0.808
Fold 1 (P2)	0.735	0.281	0.293	0.580
Fold 2 (P3)	0.692	0.048	0.106	0.566
Mean ± Std	0.693±0.041	0.285±0.239	0.275±0.161	0.652±0.136

**Table 8:** Ablation on the source of the pretrained backbone. All other components of the pipeline are identical between the two configurations. Values are mean ± sample standard deviation over n=3 LOPO folds.

Backbone Initialization	Mean Test AUC-ROC	Per-Fold Std
ImageNet (baseline)	0.656	0.084
SIPaKMeD (this work)	0.693	0.041
Absolute improvement	+0.037	−0.043
Relative improvement	+5.7%	−51.2%

**Table 9:** Five-fold patient-level cross-validation metrics.

Fold	n	$n^{+}$	AUC-ROC	AP	Best F1	Brier
0	15,183	1815	0.803	0.294	0.385	0.194
1	69,370	4802	0.530	0.096	0.164	0.072
2	9802	406	0.750	0.101	0.184	0.312
3	947	2	0.726	0.006	0.014	0.078
4	38,402	156	0.660	0.015	0.048	0.033
Mean ± Std	—	—	0.694±0.105	0.102±0.119	0.159±0.146	0.138±0.116

**Table 10:** Aggregate mean test AUC-ROC under the 5-fold patient-level cross-validation, with B=1000 patient-aware bootstrap 95% confidence interval. The ImageNet reference is the point estimate from the controlled three-fold ablation reported in Table 8.

Backbone Initialization	Mean AUC	95% CI	Source
SIPaKMeD (this work)	0.694	[0.681,0.705]	5-fold bootstrap
ImageNet (reference)	0.656	—	3-fold ablation (Table 8)

**Table 11:** Classical machine-learning baselines under the 5-fold patient-level cross-validation, on the four folds with non-degenerate positive support. Mean and standard deviation are computed with n−1 degrees of freedom over the four evaluable folds. The CNN row reports the SIPaKMeD-pretrained ResNet-50 on the same four folds for direct comparison.

Classifier	Mean AUC-ROC	Mean AP
Logistic Regression ($\ell_{2}$, handcrafted)	0.736±0.135	0.168±0.149
XGBoost (handcrafted)	0.713±0.158	0.193±0.226
ResNet50 (SIPaKMeD-pretrained, this work)	0.686±0.111	0.105±0.131

**Table 12:** Patient-level aggregation case study. “GT prev.” is the ground-truth fraction of HPV-positive patches in the fold; “Mean”, “Top-10”, and “P95” are three aggregation rules applied to the patch-level probabilities predicted by the SIPaKMeD-pretrained model.

Fold	Patients	Patches	GT Prev.	Mean	Top-10	P95
0	4	15,183	0.120	0.382	0.876	0.735
1	4	69,370	0.069	0.070	0.996	0.271
2	4	9802	0.041	0.510	0.978	0.885
3	4	947	0.002	0.171	0.594	0.538
4	3	38,402	0.004	0.091	0.755	0.462

## Data Availability

The dataset used in this study contains sensitive patient-derived imaging data and is not publicly available due to ethical and privacy restrictions. Reasonable requests for access to the anonymized data for the purpose of scientific reproducibility may be directed to the corresponding author and will be evaluated subject to institutional approval. The source code of the pipeline is available from the corresponding author upon reasonable request. The SIPaKMeD dataset used for pretraining is publicly available [12].

## Abbreviations

The following abbreviations are used in this manuscript:

AUC	Area Under the Curve
AP	Average Precision
CIN	Cervical Intraepithelial Neoplasia
CNN	Convolutional Neural Network
CV	Cross-Validation
H&E	Hematoxylin and Eosin
HPV	Human Papillomavirus
HSIL	High-grade Squamous Intraepithelial Lesion
HSV	Hue–Saturation–Value Color Space
IHC	Immunohistochemistry
LOPO	Leave-One-Patient-Out
LSIL	Low-grade Squamous Intraepithelial Lesion
MIL	Multiple Instance Learning
OD	Optical Density
ROC	Receiver Operating Characteristic
ROI	Region of Interest
SVD	Singular Value Decomposition
WSI	Whole Slide Image
